# A Fumonisin Prevention Tool for Targeting and Ranking Agroclimatic Conditions Favoring Exposure in French Maize-Growing Areas

**DOI:** 10.3390/toxins13030214

**Published:** 2021-03-16

**Authors:** Agathe Roucou, Christophe Bergez, Benoît Méléard, Béatrice Orlando

**Affiliations:** Arvalis—Institut du Végétal, Station Expérimentale, 91720 Boigneville, France; christophe.bergez@free.fr (C.B.); b.meleard@arvalis.fr (B.M.); b.orlando@arvalis.fr (B.O.)

**Keywords:** maize, *Fusarium verticillioides*, fumonisins, climate, agricultural practices, water deficit, tool management

## Abstract

The levels of fumonisins (FUMO)—mycotoxins produced by *Fusarium verticillioides—*in maize for food and feed are subject to European Union regulations. Compliance with the regulations requires the targeting of, among others, the agroclimatic factors influencing fungal contamination and FUMO production. Arvalis-Institut du végétal has created a national, multiyear database for maize, based on field survey data collected since 2003. This database contains information about agricultural practices, climatic conditions and FUMO concentrations at harvest for 738 maize fields distributed throughout French maize-growing regions. A linear mixed model approach highlights the presence of borers and the use of a late variety, high temperatures in July and October, and a water deficit during the maize cycle as creating conditions favoring maize contamination with *Fusarium verticillioides*. It is thus possible to target a combination of risk factors, consisting of this climatic sequence associated with agricultural practices of interest. The effects of the various possible agroclimatic combinations can be compared, grouped and classified as promoting very low to high FUMO concentrations, possibly exceeding the regulatory threshold. These findings should facilitate the creation of a national, informative and easy-to-use prevention tool for producers and agricultural cooperatives to manage the sanitary quality of their harvest.

## 1. Introduction

France is the second largest producer of maize in Europe, with a harvest of more than 12.5 million tons from an area of 1.4 million hectares in 2019 [1]. Maize is commonly contaminated with mycotoxins produced by fungi. The presence of these mycotoxins is associated with economic losses due to decreases in crop yields, a loss of crop value, effects on animal productivity, and health impacts. Fumonisins (FUMO) are among the most important mycotoxins affecting the sanitary quality of maize in the European Union (EU) [2]. FUMO constitute a group of at least 15 mycotoxins, the most common of which are fumonisins B1 (FB1) and B2 (FB2). In France, these toxins are produced principally by *Fusarium verticillioides,* from the genus *Gibberella*. FUMO decrease grain yield and quality, and are toxic to humans and animals; their presence in maize is, therefore, a major health and safety concern [3]. Like many other countries, the EU has imposed regulations for the permissible levels of FUMO in feed and food, due to the health risk associated with their consumption (Commission Regulation 1126/2007). The maximum permissible level of FUMO in unprocessed maize intended for human consumption is currently 4000 µg/kg. Technological advances in crop production methods and in the postharvest handling and treatment of maize are increasing the ability of maize producers and processors to decrease the likelihood of mycotoxins reaching unsafe levels in their produce [4]. Plant breeding has been seen as the safest way to reduce FUMO contamination in maize crops, given the uncontrollable nature of the climate [5,6]. However, no maize genotypes highly resistant to infection with *F. verticillioides* or to FUMO contamination have yet been produced. Moreover, genetically modified organism (GMO) cultivation is forbidden in France [7], so there is a need for management strategies to prevent contamination with this mycotoxin. FUMO management requires a multifaceted approach including a preharvest strategy for preventing the production of FUMO or mitigating their effects [4]. Such a preharvest strategy requires the identification of risk factors influencing the contamination and production of FUMO by *F. verticillioides* in the field, which can then be targeted.

*Fusarium verticillioides* infection and growth, and FUMO production, result from the complex interaction of several agronomic and climatic factors. The agronomic risk factors include kernel damage due to borer insects, which promotes FUMO accumulation [5]. Indeed, the insects provide a route of entry for the fungus, distributing fungal propagules as they feed and proliferate, and creating wounds that can then be infected by microconidia or mycelia already present on the ear tissues [8,9,10]. In the central United States and Europe, kernel infection is closely correlated with insect injury, due principally to *Ostrinia nubilabis*, and this appears to be one of the most important infection pathways [11,12]. The earliness of the plant variety has also been highlighted as a risk factor for FUMO contamination. Early maturing varieties may be less sensitive to FUMO contamination if the kernels mature quickly, such that kernel moisture rapidly falls below the levels favorable for *F. verticillioides* growth and sporulation [6]. The temperature is one of the most relevant environmental factors influencing fungal growth and mycotoxin production. FUMO production is greater in warmer maize-growing areas worldwide [13]. Indeed, the sporulation, germination and growth of *F. verticillioides* are optimal at 25 °C–30 °C [14,15,16], and FUMO production is optimal between 15 °C and 25 °C [17]. Maize kernels are infected by *F. verticillioides* principally via the silks [18], and flowering is, thus, one of the most sensitive periods for infection. The risk of FUMO contamination may also begin early in maize ear development, increasing as the kernels approach physiological maturity [19]. Ripening is, therefore, also a very sensitive period for contamination. For both contamination by the fungus and mycotoxin production, climatic and agronomic conditions must together create a prosperous environment for the fungus, at the right stage of development. It is therefore essential to highlight the complex interactions between agronomic and climatic risk factors during maize development, to identify risky situations in the field. A knowledge of these situations would make it possible to develop preharvest strategies in the field.

With climate change, water deficits will become increasingly frequent during the cereal growth season in Europe [20]. For American maize-growing regions, Wu et al. [13] suggested that climate change trends might result in higher preharvest levels of mycotoxins, posing both economic and health risks. In this context, several multiyear studies in maize-growing areas around the world (Nebraska, Mississippi, Poland and South Africa) have shown that levels of *F. verticillioides* and FUMO contamination are highest in nonirrigated fields and in situations in which rainfall is below usual levels [3,21,22,23]. Hypotheses have been put forward to explain the higher FUMO levels on the basis of the impact of drought stress on the plant or fungus growth. Miller et al. [10] suggested that drought stress may compromise host plant defenses against pathogens. Others have suggested that stressful conditions may stimulate mycotoxin production as a means of adapting fungal growth [5,24]. Alternatively, it has been suggested that dry conditions during flowering may favor the movement of insects within ears, increasing the likelihood of FUMO contamination [25]. The effects of high temperatures and drought stress may be confounded, as excessive heat is generally accompanied by dry conditions. It can, therefore, be difficult to separate the two factors and to determine the individual effects of each. Little is currently known about the consequences of water stress for the combination of agronomic and climatic factors driving FUMO contamination in the field. Water stress may exacerbate situations that are already risky or create conditions favorable for fungal growth and mycotoxin production at particular times in plant development. It is therefore necessary to evaluate the impact of water stress on the various agroenvironments encountered in French maize-growing regions. There is also a need to identify periods of high risk during plant development, to facilitate the adaptation of agronomic practices.

FUMO production has been observed during the postharvest period, in adverse storage conditions [26], but integrated postharvest approaches involving drying and storage management has been shown to prevent postharvest FUMO contamination effectively [27]. It is important to separate contaminated batches before harvest. The use of a prevention strategy in the field can be useful in this respect, making it possible to target the maize fields most likely to exceed the regulatory threshold for FUMO and separate them from the rest. The objective of this work was, therefore, to identify the combinations of risk factors promoting high FUMO concentrations in maize, possibly exceeding regulatory thresholds, which could then be targeted for the creation of prevention tools for use in the field.

## 2. Results

### 2.1. Agronomic and Climatic Factors Influencing FUMO Concentrations

An analysis of variance (ANOVA) on FUMO concentrations was performed at the national scale in France. The earliness of the maize variety sown, and the presence of borers were the only factors found to have significant effects among agronomic factors (*p*-value < 0.001, for both).

**Figure 1:**
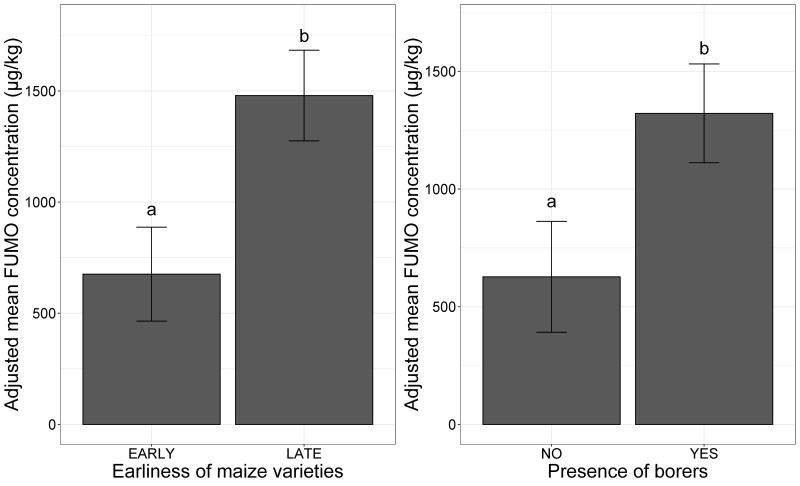
Mean Fumonisins (FUMO) concentration adjusted for agronomic factors, in maize. (**a**) Mean adjusted FUMO concentration according to the presence or absence of boring insects; (**b**) mean adjusted FUMO concentration according to the earliness of the maize variety. The two categories for earliness (EARLY, LATE) and for borer presence (NO, YES) are shown on the *x*-axis. The adjusted mean FUMO concentration obtained with a mixed linear model (lmer(FUMO ~ factor + (1|Year)) applied to a database of 738 observations is plotted on the *y*-axis. Different letters (a and b) above the bars indicate significant differences at *p*-value < 0.001 in ANOVA test for each variable.

Early varieties were significantly less susceptible to FUMO contamination than late varieties (Figure 1a). The presence of borers in the agricultural plots significantly increased the adjusted mean FUMO concentration (Figure 1b).

This multiyear study of the effects of more than 400 climatic variables on FUMO contamination risk during the maize development cycle revealed a main effect of temperature, particularly for the mean temperatures of two key months: July and October (*p*-value *<* 0.001 for both, ANOVA). In France, July is the month in which maize flowering occurs, and October is the month in which ripening occurs. Two temperature thresholds, the medians of all the values measured at the scale of France for 17 years, were used to transform the two quantitative variables into two qualitative variables (Table 1). The two categories differentiating a “hot” month from a “cool-to-normal” month for these two months of the year were defined on the basis of their range values in relation to the seasonal values (Table 1). Using these categorical climatic factors, the adjusted means were calculated for each category and compared (Table 1).

**Figure 2:**
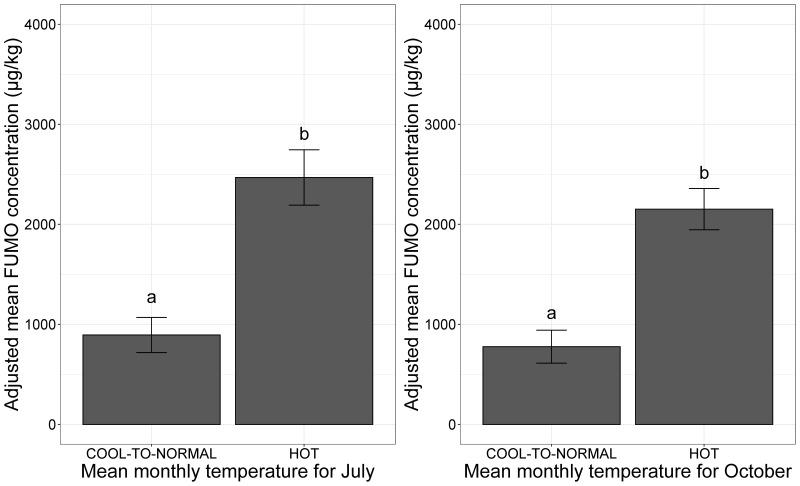
Mean adjusted FUMO concentration according to mean monthly temperature at flowering and at maturity. (**a**) Adjusted mean FUMO concentrations by July mean monthly temperature; (**b**) adjusted mean FUMO concentrations by October mean monthly temperature. Mean monthly temperature category is indicated on the *x-*axis for both plots (COOL-TO-NORMAL, HOT). The adjusted mean FUMO concentrations obtained by applying the mixed linear model (lmer (FUMO ~ July/October mean monthly temperature + (1|Year)) to a database of 738 observations are plotted on the *y*-axis. Different letters (a and b) above bars indicate significant differences at *p*-value < 0.001 in ANOVA test for each variable.

The mean monthly temperatures in July and October had significant effects on the risk of FUMO contamination in maize at French national scale (*p*-value < 0.001 for both climatic factors, ANOVA) (Figure 2).

### 2.2. Association of Risk Factors for FUMO Contamination

**Figure 3:**
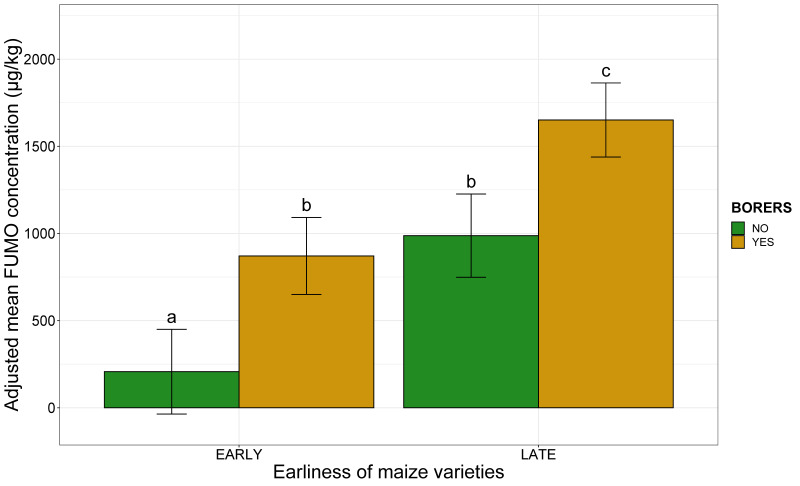
Adjusted mean FUMO concentrations, according to maize variety earliness and the presence of borers. The earliness of the maize variety is plotted along the *x*-axis (EARLY, LATE). The adjusted mean FUMO concentration obtained by applying the mixed linear model (lmer(FUMO ~ Earliness*Borers + (1|Year)) to a database of 738 observations is plotted on the *y*- axis. Green bars correspond to plots without borers, whereas yellow bars correspond to plots with borers. Different letters (a, b and c) above the bars indicate significant differences at *p*-value < 0.05 in Tukey’s multiple comparison test.

The combination of the two agronomic factors had a significant impact on FUMO contamination (R^2^ = 0.06, *p*-value < 0.001). Adjusted mean FUMO concentration increased as soon as one of the two factors was in the “at risk” category, the modality related to higher FUMO content, and this increase was larger if both factors were in the “at risk” category (Figure 3). The plots planted with late maize varieties and infested with borers were at the highest risk, with an adjusted mean FUMO concentration of 1651 µg/kg (Figure 3).

**Figure 4:**
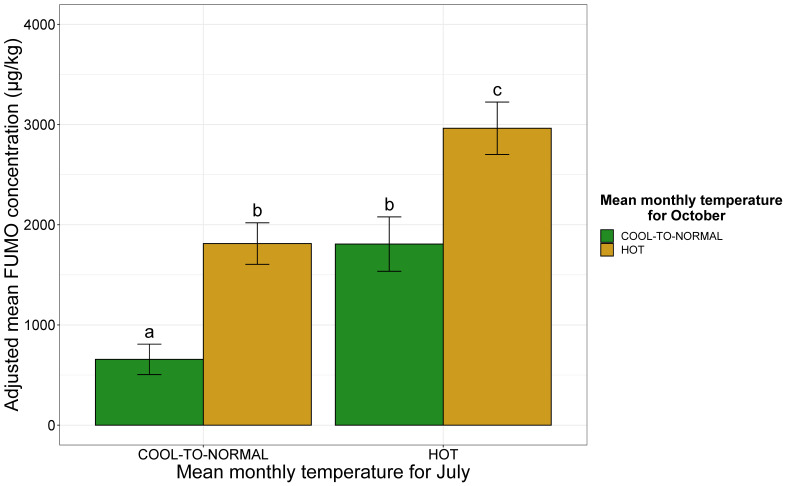
Adjusted mean FUMO concentration according to mean monthly temperatures in July and October. The mean monthly temperature for July is shown on the *x*-axis (COOL-TO-NORMAL, HOT). The adjusted mean FUMO concentration obtained by applying the mixed linear model (lmer(FUMO ~ July monthly temperature*October monthly temperature + (1|Year)) to a database of 738 observations is shown on the *y*-axis. Green bars correspond to observations for cool-to-normal temperature Octobers, whereas yellow bars correspond to data for hot Octobers. Different letters (a, b and c) above the bars indicate significant differences at *p*-value < 0.05 in Tukey’s multiple comparison test.

The combination of the two climatic factors had a significant effect on FUMO contamination (R^2^ = 0.15, *p*-value < 0.001). The adjusted mean FUMO concentration increased if either of the two months was considered as “hot” (Figure 4). High temperatures during maize flowering and ripening are the principal risk factors for FUMO production, and high temperatures in both these months led to adjusted mean FUMO concentrations exceeding 3000 µg/kg (Figure 4).

### 2.3. The Presence of a Water Deficit Accentuates the Effect of Agronomic and Climatic Conditions on the Risk of FUMO Contamination in Maize

The individual effect of a water deficit on the risk of FUMO contamination was not significant in the nationwide multiyear study (*p*-value > 0.05, ANOVA).

**Figure 5:**
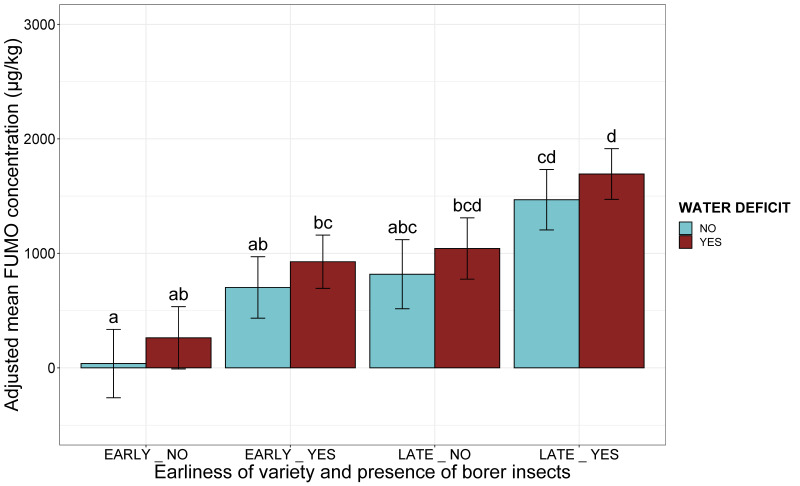
Adjusted mean FUMO concentration according to the earliness of the variety, the presence of borers and water deficit. The two agronomic factors are represented as a composite variable on the *x*-axis (EARLINESS_BORERS). The adjusted mean FUMO concentration obtained by applying the mixed linear model (lmer(FUMO ~ EARLINESS_BORERS*WATER DEFICIT + (1|Year)) to a database of 738 observations is plotted on the *y*-axis. Blue bars correspond to observations for the absence of a water deficit, whereas red bars correspond to data for the presence of a water deficit. Different letters above the bars indicate significant differences at *p*-value < 0.05 in Tukey’s multiple comparison test.

The presence of a water deficit in association with the agronomic risk factors slightly (by 224 µg/kg), but not significantly increased FUMO content (*p*-value > 0.05, test ANOVA, Figure 5). Including the presence or absence of water stress in the analysis did not increase the proportion of the variability explained by agronomic conditions (R^2^ = 0.06, *p*-value < 0.001). The riskiest combination consisted of a water deficit in a late variety infested with borers, with an adjusted mean FUMO concentration of 1693 µg/kg (Figure 5).

**Figure 6:**
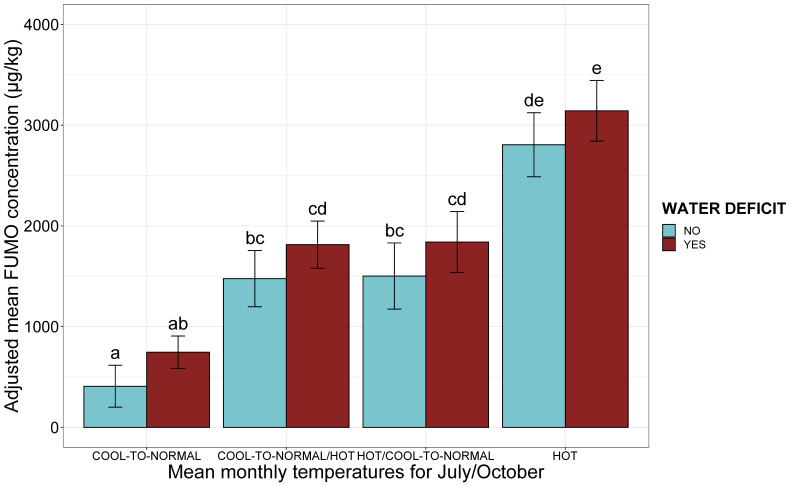
Adjusted mean FUMO concentration according to mean monthly temperatures at flowering and maturity and the presence or absence of a water deficit. A composite variable for the temperatures in July and October (July/October when different) is shown on the *x*-axis. The adjusted mean FUMO concentrations obtained by applying the mixed linear model (lmer(FUMO ~ July_October monthly temperature*WATER DEFICIT + (1|Year)) to a database of 738 observations are shown on the *y*-axis. Blue bars correspond to observations for the absence of a water deficit, whereas red bars correspond to data for the presence of a water deficit. Different letters above bars indicate significant differences at *p*-value < 0.05 in Tukey’s multiple comparison test.

When combined with climatic risk factors, the individual effect of water deficit was of borderline significance (*p*-value = 0.06, ANOVA). Whatever the temperature in July or October, the presence of a water deficit was associated with a mean increase in FUMO concentration of 341 µg/kg (Figure 6). The addition of water stress to the model with climatic variables did not increase the proportion of the variability explained by climatic conditions (R^2^ = 0.15, *p*-value *<* 0.001). The riskiest combination was a combination of a water deficit with a hot July and a hot October, with an adjusted mean FUMO concentration of 3143 µg/kg (Figure 6).

### 2.4. Combinations of Categories for the Agronomic and Climatic Factors Can Create Definitions Determining Whether the Regulatory Limits for FUMO in Maize in the EU Are Respected

**Figure 7:**
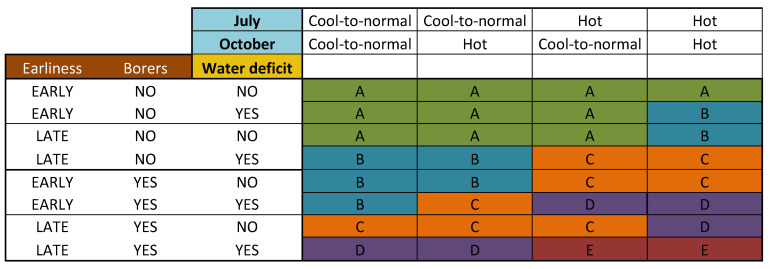
Grid based on the multiyear data showing the impact of combinations of risk factors on FUMO content in maize. Agronomic factors (earliness and borers) and water deficit are represented on the left of the grid. Climatic factors (mean monthly temperatures in July and October) are represented at the top of the grid. The various intersections were grouped into a common factor ITK. Their adjusted means were obtained by applying the mixed linear model (lmer(FUMO ~ ITK + (1|Year)) to a database of 738 observations. These means were compared, grouped if not statistically different and then assigned to FUMO risk class (A to E), which are shown in different colors. In ascending order, A corresponds to a very low risk (green), B to a low risk (blue), C to a moderate risk (orange), D to a high risk (purple) and E to a critical risk (red).

The association of the agronomic and climatic risk factors described above accounted for 19% of the variability of FUMO concentration observed over a period of 17 years at the national scale in France (R^2^ = 0.19, *p*-value < 0.001). The grid based on the multiyear data shows the possible combinations of the categories of these variables in the field (Figure 7). The agroclimatic combinations with similar effects on FUMO contamination were grouped together in five different groups. A risk class for FUMO contamination was assigned to each category, extending from a very low risk (A) to a critical risk (E) (Figure 7).

Class A, corresponding to a very low FUMO risk, was the most represented class (Figure 7). However, risk class gradually increased with combinations of different agronomic and climatic risk factors (Figure 7). The high and critical risk classes corresponded to favorable agronomic conditions (i.e., a late variety and the presence of borers) combined with at least one of the critical climatic sequences (hot July and/or October, and/or water deficit) (Figure 7). In general, water stress tends to increase the risk class (Figure 7).

**Figure 8:**
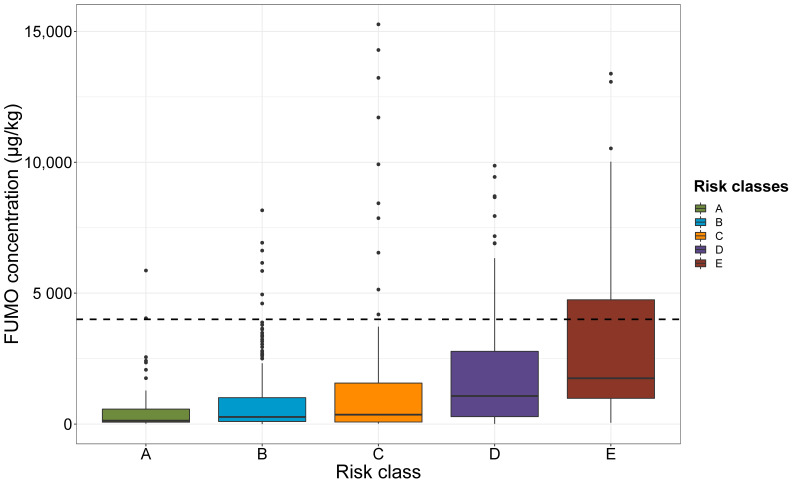
FUMO concentration according to risk class. The risk classes for FUMO contamination are shown along the *x*-axis, in ascending order of risk, from left to right (A to E). The FUMO concentration of the 738 plots measured are shown on the *y*-axis. Colors distinguish the different risk classes: green for A, blue for B, orange for C, purple for D and red for E. Dashed line represents the EU maximum limit for FUMO in food for human consumption (4000 µg/kg).

The results of the validation of this multiyear grid are shown in Figure 8. FUMO contamination was positively related to risk class (*p*-value < 0.001, ANOVA). Over the 17-year period for which data were analyzed, the mean, median, first and third quartiles of FUMO concentration clearly increased from Class A to E (Figure 8). Extreme values were observed for each risk class (Figure 8). For Class D, 11% of the values exceeded the maximum limit of 4000 µg/kg allowed. The percentage of values exceeding this limit increased to 35% for class E (Figure 8).

The FUMO risk classes created accounted for 10% of the variability in FUMO concentration observed over the 17 year period (Table 2). This percentage varied from year to year, reaching up to 50% (Table 2).

## 3. Discussion

We used a unique database containing the data from 738 agricultural farm fields in French maize-growing regions collected over a period of 17 years to explore and evaluate agronomic and climatic risk factors, and the effect of water stress on FUMO contamination in the field at harvest. Such associations have been little explored to date because preharvest strategies in maize fields mostly involve the use of agronomic tools to adapt the technical itinerary [14,28]. The impact of water stress, favoring contamination with FUMO, has been mentioned by several studies [10,29], but none quantified this effect. Most of the prevention strategies for reducing the risk of FUMO contamination are based on genetic research to identify plant resistance to *F. verticillioides* contamination and FUMO production [30]. GMOs are banned in France and therefore cannot be used as a tool against FUMO contamination. However, preventive measures that can be applied while the crop is growing in the field are the first and most crucial step towards producing maize grains with acceptable levels of FUMO contamination for the various cereal market outlets [14]. In this context, we provide a FUMO prevention tool based on the agronomic and climatic conditions encountered in French maize-growing areas (summarized in Figure 7). Each situation is associated with a risk class, from very low to critical.

We identified the use of late varieties and the presence of borers as agronomic factors favoring contamination. These factors have already been evaluated as risk factors for preharvest FUMO contamination in several studies [9,11,21,31,32]. In particular, the presence of borers was included in a previous prevention matrix created by Arvalis-Institut du végétal in 2007 [28]. Several studies have proposed the development of preharvest strategies based on the use of agronomic practices to limit FUMO contamination, including the use of an appropriate selection of maize hybrids and crop density, and avoiding late sowing and harvest dates, for example [14,27,30,33,34]. All of these proposals were developed on the basis of field experiments. Here, we assessed these factors in real production conditions, at a nationwide scale. Only the presence of borers and one characteristic of the crop (earliness) were identified as significantly associated with the risk of contamination. Our findings confirmed the contribution of these factors to the observed variability of FUMO content in the field over 17 years at French national scale. However, these factors had a smaller impact than climatic conditions. The climate is often unpredictable and difficult to modulate as a prevention tool. However, two critical periods in the maize growing season favoring *F. verticillioides* infection and FUMO contamination have been identified and studied in detail: flowering and kernel drying. Higher temperatures and drier weather during flowering, and higher temperatures during kernel maturation have been shown to increase FUMO content at harvest [23,35,36,37,38,39]. Our findings confirm this influence of temperature on FUMO contamination during these two periods. We went further by defining a threshold above which monthly temperatures can be considered “hot” in France. This made it possible to simplify complicated climatic variables by transforming them into more easily usable variables. Another climatic factor has long been suggested to influence the risk of FUMO contamination in maize: drought [10,29]. In 1996, Tardieu defined drought as a prolonged period without precipitation that can result in a decrease in soil water content, thereby causing water deficit in the plant [40]. In this context, water stress can be defined either in terms of external water levels around the plant (in the soil or air), or in terms of the internal water in the plant tissues, measured directly or indirectly, via evaluations of the physiological processes responding to the water levels in the plant [41], such as leaf expansion [42]. In this study, we had access only to external variables linked to the water stress. We chose to focus on soil water availability, which is related to rainfall levels and soil type and can be used to define water stress in farm fields at the national scale. Our findings confirmed that the risk of FUMO contamination was higher in the event of water stress [5,13,29], but only when the agroclimatic situation was already favorable. Our findings thus confirm the climatic and agronomic risk factors already identified in previous studies. However, we went further by using these risk factors to construct simple, easy-to-use agronomic and climatic explanatory variables and to create a FUMO prevention tool.

The multiyear and national grid ranks associations between agronomic and climatic conditions from very low to critical in terms of FUMO risk in French maize-growing areas. In the first decade of this century, two European tools were created to help farmers, agricultural cooperatives and processors to manage the FUMO contamination of maize grain in northern Italy. First, Battilani et al. [43] created a logistic regression model capable of identifying cropping systems for which the EU regulatory limit was likely to be exceeded. This model was created with data from field surveys performed from 2002 to 2007 [43]. It highlighted specific agronomic factors associated with the risk of contamination: preceding crop, maturity class (mean number of days from emergence to maturity), sowing week, nitrogen levels, harvest week and grain moisture [43]. Maiorano et al. [14] then created an agronomic decision tool based on the concept of agronomic exposure to fumonisin risk. This tool measures the capacity of a crop management system to control the FUMO contamination of maize grains [14]. Based exclusively on agronomic factors, planting and harvest dates and chemical treatment, the authors were able to classify the different management decision combinations from low to critical FUMO risk for Italian farm fields from 2003 to 2005 [14]. In both studies, the authors confirmed the large contribution of the cropping system to FUMO contamination in maize. The earliness of the variety and the presence of borers provide less information about crop management than the factors included in these two tools. However, they were the only two factors that had a significant effect on the occurrence of FUMO contamination in France over the 17 years studied. One possible reason for this is the tremendous diversity of management systems observed in our field surveys, which may have made it much harder to target more specific systems. Despite the good results obtained, differences between observations and predictions were observed for specific years [14,43]. Unusual weather conditions, particularly during flowering, were identified as the likely cause of these differences. Battilani et al. [43] considered the lack of meteorological data to be a weakness of their tool. Moreover, both authors described water deficit as a factor affecting FUMO contamination [14,43]. In this context, our tool can combine certain climatic conditions, and the notion of water stress, with agronomic practices. The tool created by Battilani et al. [43] was able to account for 60% of the variability of FUMO concentration observed in northern Italy over a period of six years. Our selection of variables accounts for 19% of the variability over the whole of France over a period of 17 years. This difference may reflect a difference in the scales of the two studies: the north of Italy for 6 years versus nationwide in France for 17 years. Our tool is new and was created for use at the national scale, based on a combination of agronomic and climatic conditions and water stress influence.

The prevention tool assigned a risk class to each farm field over 17 years at national scale. It was developed and validated with data and information from agricultural farm fields in the various French maize-growing regions. Some plots considered to be at very low to low risk for FUMO contamination actually had higher concentrations than predicted in our study, but most of the plots were well characterized over the 17-year period. Studying broader situations, such as the different agronomic and environmental conditions encountered, would be complicated [14]. Given the number of regions studied, differences in both maize management systems and local climate would have to be taken into account. By reducing the number of categories for each factor and transforming quantitative variables into qualitative ones, we were able to simplify the grid, to help farmers to adapt it to the conditions in their own farm fields. For example, the characterization of temperatures as “hot” or “cool-to-normal” could be left to the discretion of farmers and would therefore depend on the location of their farm fields. This approach makes it possible for farmers and agricultural cooperatives to manage the variables themselves and should help to reduce regional effects. The association of a risk class with each combination of simplified categories of the factors considered decreased the proportion of the variability explained to 10% over the 17 years, due to a loss of information: a decrease in the number of possible combinations from 32 to 5. We chose to decrease the number of categories, to make it possible to combine similar field situations and to make the tool easier to interpret. Shelby et al. [35] found that dry weather was most likely to affect FUMO production at or just before pollination. With our water stress calculations, we were unable to focus on flowering time. Further studies are needed to determine the differences in the impact of water stress at different plant stages. Our results show that at least one period of water stress during the maize crop cycle can increase the risk in situations already at risk of FUMO contamination in maize fields. Our tool requires additional testing by the various stakeholders of the maize sector (farmers, agricultural cooperatives), to determine whether it meets their expectations and whether and how it could be improved. Against a background of increasingly strict EU regulations for mycotoxins, it will be essential to have accurate tools for preventing the contamination of batches of maize grain. The good results obtained with this tool for the 17 years studied at nationwide level in France suggest that the approach followed here for the development of this tool could be used in other countries and for other mycotoxins.

One perspective of this work would be the use of this tool in other countries of the EU. However, the differences in systems and environments are already large enough at the national scale in France and would be even larger between different European countries. In Spain, Ruiz et al. [44] showed that location was the principal environmental source of variation affecting the occurrence of FUMO contamination in maize. In Italy, Maiorano et al. [14] suggested that this major role of location was due to environmental effects. It would probably be difficult to use our risk grid in its current state in other European countries, but the grid could be modified to deal with other realities in the field. A similar approach could be used to select agronomic practices and meteorological conditions and to make the suitable adaptations, particularly as concerns temperature thresholds during flowering and ripening. It would also be possible to expand the grid to consider the co-contamination of maize with other mycotoxins. Indeed, different fungi can co-contaminate the same maize plant and produce different mycotoxins, such as FUMO (produced by *F. verticillioides)* and aflatoxins (produced by *Aspergillus flavus)*. Scientific interest in the biological effects of mycotoxin mixtures is increasing. Methods were developed to detect mycotoxin at the single kernel level and study the potential co-occurrence of FUMO and aflatoxins [45]. Indeed, the nature of the relationship between the two mycotoxins needs to be further studied [46,47]. The balance between them differs between regions and depends on agricultural practices, but is also partly governed by climate and weather [47]. This co-occurrence of FUMO with other mycotoxins in maize is problematic for the creation of accurate prevention tools, as the presence of these other mycotoxins may affect FUMO content. Further studies are required to incorporate this balance between co-contaminants into the FUMO prevention tool.

## 4. Conclusions

In this study, a national multiyear field survey was performed in French maize-growing areas, to study the relationship between FUMO contamination and agroclimatic environments. Five agronomic and climatic factors were identified: the presence/absence of borers, the earliness of varieties, the temperature during July and October, and the presence of a water deficit during cycle development. The effects of their various categories were analyzed, and the combinations of these categories were grouped and ranked, for classification of the risk in the field from very low to high. The riskiest situation was late maize varieties, infested with borers, in farm fields with hot temperatures in July and October, and water stress. These risk factors have already been identified individually in several studies, but not in combination. Most prevention tools assess crop system managements. Our FUMO prevention tool includes both agronomic and climatic factors, and also integrates the amplifying effect of water stress. The transformation of quantitative climatic variables into qualitative variables made it possible to integrate more accessible climatic sequences in a field tool created for all stakeholders in the sector. Our study made it possible to combine the various crop systems and climatic conditions occurring in France into a single prevention tool. Improvements in our understanding of the biotic relations between co-contaminants are now required, to determine the balance between mycotoxins, which could be incorporated into future prevention tools.

## 5. Materials and Methods

### 5.1. Multiyear Field Surveys at the French National Scale

Maize is the second most important cereal crop in France after wheat, with an annual mean of 1.5 million hectares sown. From 2003 to 2019, 738 samples were collected at harvest from 738 farm fields of maize as part of our national mycotoxin monitoring of the maize harvest (Table 3). In that context, a call for volunteers was issued among farmers in the French maize-growing areas. The choice and number of farm fields chosen took into account (1) the relative importance of each location to French maize production and (2) the relative importance of maize to French cereal production. The annual variation of farm fields studied depended on our internal budget available to finance this study.

**Figure 9:**
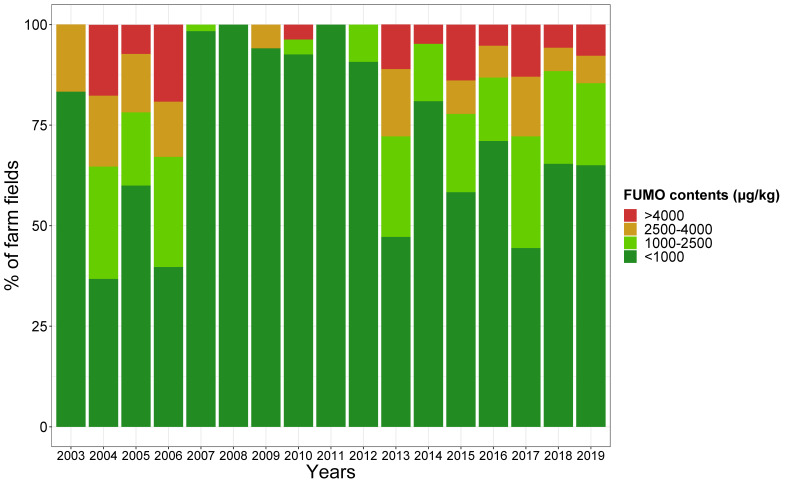
Annual distribution of farm fields into FUMO concentration classes. Years are shown in chronological order. Four concentration classes were defined: <1000 µg/kg (dark green), 1000–2500 µg/kg (light green), 2500–4000 µg/kg (orange) and >4000 µg/kg (red). The number of samples per year is indicated in Table 3.

The FUMO contents of the farm fields studied are summarized in Figure 9. Over the study period, FUMO concentrations were high in some years (2004, 2017, etc.) and very low in others (2008, 2011, etc.).

**Figure 10:**
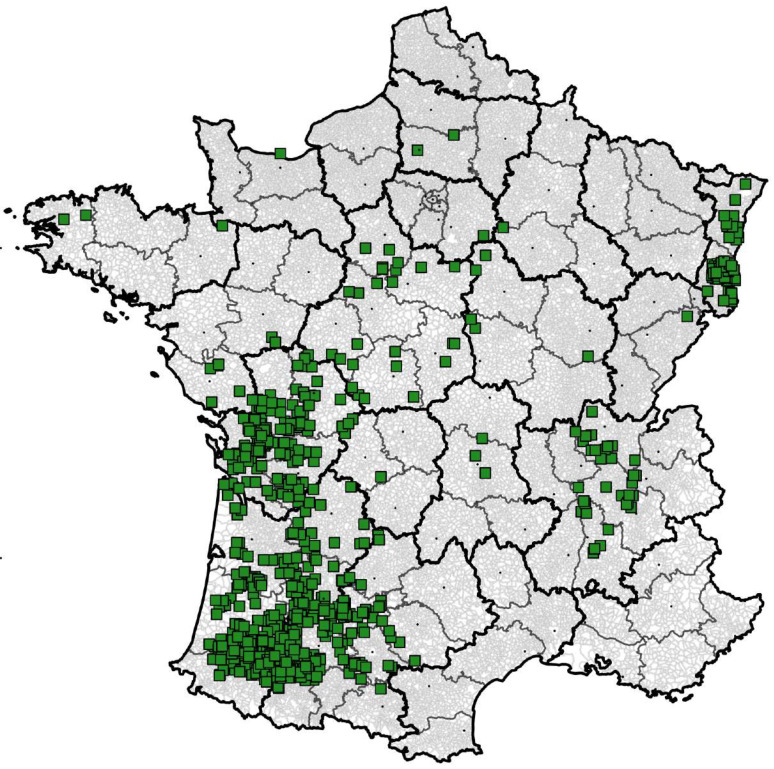
Geographic distribution of field samples in France over the study period.

The spatial distribution of the farm fields is described in Figure 10 and is representative of the main French maize-growing areas. The spatial distribution was similar in all 17 years of the study.

### 5.2. Sample Collection

At harvest, farmers were asked to prepare samples, according to the following instructions: (a) avoid sampling field margins, (b) avoid static grain sampling, and (c) sample moving grains during three different periods of emptying of the combine harvester. Three different subsamples, each weighing at least 1 kg, were therefore collected manually from the moving grains during harvest. These three subsamples were then combined to obtain a 3 kg final sample from each farm field.

### 5.3. Sample Preparation for Analysis

All the grain samples were cleaned with a laboratory cleaner and separator (MINI-PETKUS 100 and 200, PETKUS Technologie GmbH, Rohr, France) to remove all impurities from the kernels. We then took 1.5 kg of cleaned and homogeneous sample for analysis. This sample was ground in a laboratory hammer mill fitted with a 1 mm screen (TITAN 2000, F.A.O., Vitré, France).

### 5.4. Fumonisin Quantification

From 2003 to 2013, fumonisins B1 and B2 (FB1 and FB2) were analyzed by liquid chromatography with photometric detection (HPLC-UV) at two accredited laboratories in France. Samples were randomly shipped to either laboratory. For the first laboratory, the limits of detection for FB1 and FB2 were 10 and 30 µg/kg, respectively, and the corresponding limits of quantification were 30 and 100 µg/kg. For the second laboratory, the limits of detection for FB1 and FB2 were 71 and 76 µg/kg, respectively, and the corresponding limits of quantification were 256 and 282 µg/kg.

From 2014 to 2019, FB1 and FB2 were analyzed by liquid chromatography-tandem mass spectrometry at the second accredited laboratory in France. The limits of detection for FB1 and FB2 were 5 and 25 µg/kg, respectively, and the corresponding limits of quantification were 10 and 50 µg/kg.

In our study, for all FUMO contents below the limit of detection, we assigned a value corresponding to half the detection limit. Similarly, for values below the limit of quantification, we assigned a value corresponding to half the quantification limit.

### 5.5. Agronomic Factors

For each farm field, the farmers were asked to complete a questionnaire developed by Arvalis-Institut du végétal with items concerning agronomic parameters (including, in particular, the variety grown and whether or not borer insects were present), location and soil type.

For earliness, the various groups were fused to create a dichotomous variable, with varieties considered to be “Early” (half early c1 dentate cornea, half early c2 toothed, early and very early) or “Late” (semi late, late and very late).

### 5.6. Climatic Factors

The town and zip code of each farm field were used to obtain the Lambert coordinates. We then used this spatialization to obtain meteorological data from the nearest weather station (Arvalis-Institut du végétal or Météo France). The weather variables used were based on daily parameters calculated from spatialized climatic data from nearly 700 weather stations distributed throughout mainland France [48].

More than 400 parameters relating to temperature, rainfall, and frost days, for example, were selected based on field expertise and literature, and then studied. First of all, climatic conditions were targeted as favorable ones for *F. verticillioides* growth and propagation, but also linked to a greater sensitivity of the maize plant. Then, these variables were calculated over specific periods of the plant cycle (calendar or in relation to sensitive stage as flowering).

### 5.7. Water Deficit

For each agricultural plot, the meteorological data, such as temperature and rainfall (P), were obtained from the nearest weather station. For water balance, Arvalis-Institut du végétal has developed a water balance model, which can distinguish between evaporation from the first 10 cm of soil and transpiration from the plant [49]. This model was used to estimate maximal transpiration through vegetation (Tv) daily, as a function of leaf area and climatic demand via evapotranspiration, to evaluate the precipitation stored in the upper layers of the soil and to estimate surface runoff (R), and soil evaporation (Es).

The available transpirable soil water (ATSW_t_) was calculated at a given time, *t*. ATSW_t_ is equal to the amount of water accessible in the soil at the start of the season (TTSW or total transpirable soil water) plus the water from P. The water lost to Tv, Es and R was then subtracted.

TTSW was estimated according to the type of agricultural soil, according to the experimental results obtained by Arvalis-Institut du végétal.

ATSW_t_ was calculated as follows:

ATSW_t_ = TTSW + P − Tv − Es − R, (1)

The ratio between the amount of usable soil water at time *t* (ATSW_t_) and the total amount of water in the soil at field capacity (maximal TTSW) corresponds to the fraction of the soil water usable by the plant at this time point (fraction of transpirable soil water or FTSW_t_). This variable provides information about changes in water reserves. In our study, TTSW was considered to be maximal (100) at sowing.

FTSW_t_ was calculated as follows:

FTSW_t_ = ATSW_t_/TTSW, (2)

Equations (1) and (2) were resolved from sowing to harvesting (i.e., throughout the maize crop cycle). An overall FTSW was then obtained for the entire period of crop development on each agricultural plot.

We used the FTSW data obtained for all years and all plots to define a threshold for water stress (presence or absence). The quantitative variable was transformed into a qualitative variable: water deficit (Table 4).

### 5.8. Statistical Analyses

All statistical analyses of FUMO content were based on the sum of the values obtained for FB1 and FB2.

#### 5.8.1. Selection of Climatic and Agronomic Risk Factors

A first shortlist of climatic risk factors was realized by a least absolute shrinkage and selection operator (LASSO) regression with a constraint of type L1 [50,51]. The most relevant variables were then selected using a random forest approach [52].

We realized one linear mixed model with individual and combined effects of the remaining quantitative climatic factors and agronomic variables, as fixed effects, with the year as a random effect. We performed analyses of variance (ANOVA) with the car package for R [53] to target risk factors with significant effect on FUMO content.

#### 5.8.2. Convert Climatic Quantitative Factors into Categorical Variables

The distribution of values for the two selected climatic quantitative factors (see Section 5.8.1) were observed over the 17 years. The quartiles were used to create four balanced categories for each factor. We performed two linear mixed models to test their individual effects on FUMO contamination (as fixed effects), with year as a random effect. We realized pairwise comparisons between the four categories, based on Tukey-adjusted least-squares means, with *p*-values < 0.05 considered significant, with the multcomp package for R [54]. We grouped the first two and the last two categories, which were not statistically different, to create two dichotomous variables with the median as a threshold.

#### 5.8.3. Univariate and Multivariate Analyses

Univariate analyses were firstly realized on the selected agronomic and categorical climatic factors to study individually their effects on FUMO content (Table 5). Then multivariate analyses tested combinations of risk factors on FUMO content (Table 5). Agroclimatic combinations with not statistically different effects on FUMO contamination were grouped and associated with a FUMO risk class, tested with an univariate analysis (Table 5).

To develop this linear mixed model approach, we analyzed individual risk factors and combinations of risk factors with linear mixed models, in which agronomic and/or climatic factors were treated as fixed effects, with year as a random effect (Table 5). We performed analyses of variance (ANOVA) with the car package for R to evaluate the effects of individual risk factors and combinations of risk factors on FUMO content. Adjusted means were calculated and analyzed with the emmeans package for R [55]. The statistical significance of differences between the categories of a risk factor, and between combinations of categories of different risk factors was assessed in pairwise comparisons, based on Tukey-adjusted least-squares means, with *p*-values < 0.05 considered significant, with the multcomp package for R [54].

All data processing and statistical analyses were performed with R, version 4.0.2 (R Development Core Team, 2020) [56].

## Figures and Tables

**Table 1:** Adjusted mean FUMO concentrations for each category of the two selected climatic factors and their comparison.

Climatic Factors	Mean of Mean Temperatures	Modalities	FUMO Adjusted Means (µg/kg)	Average Comparison
July, mean monthly temperature	<23 °C	Cool-to-normal	1002	a ^1^
	>23 °C	Hot	2397	b ^1^
October, mean monthly temperature	<15.7 °C	Cool-to-normal	803	a ^2^
	>15.7 °C	Hot	2352	b ^2^

^1^ Mean values in each row followed by different letter are significantly different at *p*-value < 0.001 (ANOVA); ^2^ Although these are the same letters, they are not the same groups.

**Table 2:** Significance of the FUMO risk classes created, for each year studied. For each year, *p*-values and R^2^ are provided for the combinations of risk factors in linear fixed-effect models with risk class as the fixed effect.

Year	Samples	*p*-Values	R^2^
2003	6	>0.05	0.19
2004	68	<0.01	0.12
2005	55	<0.001	0.28
2006	73	<0.01	0.16
2007	62	<0.01	0.12
2008	22	>0.05	0.08
2009	17	>0.05	−0.18
2010	27	>0.05	0.05
2011	11	NA	NA
2012	54	>0.05	−0.04
2013	36	>0.05	−0.01
2014	21	>0.05	0.09
2015	36	<0.001	0.54
2016	38	>0.05	−0.04
2017	57	<0.05	0.12
2018	52	<0.05	0.14
2019	103	<0.05	0.04
2003–2019	738	<0.001	0.1

**Table 3:** Sampling for the field survey, by year.

Years	Samples
2003	6
2004	68
2005	55
2006	73
2007	62
2008	22
2009	17
2010	27
2011	11
2012	54
2013	36
2014	21
2015	36
2016	38
2017	57
2018	52
2019	103

**Table 4:** The fraction of transpirable soil water (FTSW) threshold for the presence of a water deficit during the maize development cycle.

Water Deficit	FTSW
Absence	>0.4
Presence	<0.4

**Table 5:** The linear mixed model approach to test individual and combined effects of risk factors on FUMO content. The models are ordered according to the different stages of the process.

Linear Mixed Models	Objectives
[FUMO] = Agro_i_^1^/Clim_j_^2^ + (1|Year)	Individual effect
[FUMO] = ∑(Agro_i1_/Clim_j1_ ∗ Agro_i2_/Clim_j2_) + (1|Year)	Combination of climatic or agronomic effects
[FUMO] = ∑(Agro_i_ ∗ Clim_j_) + (1|Year)	Combination of agroclimatic effects
[FUMO] = Risk classes + (1|Year)	Individual effect of the FUMO risk class

^1^ Agro_i_ represents one of the selected agronomic variables (presence of borers, earliness). ^2^ Clim_j_ represents one of the climatic selected variables (July, October and water deficit).

## Data Availability

The data presented in this study are available on request from the corresponding author. The data are not publicly available because they were collected privately by Arvalis-Institut du vegetal.
